# Effectiveness of interventions for hypertension care in the community – a meta-analysis of controlled studies in China

**DOI:** 10.1186/1472-6963-12-216

**Published:** 2012-07-24

**Authors:** Zuxun Lu, Shiyi Cao, Yun Chai, Yuan Liang, Max Bachmann, Marc Suhrcke, Fujian Song

**Affiliations:** 1 Department of Social Medicine and Health Management, School of Public Health, Tongji Medical College, Huazhong University of Science and Technology, Wuhan, Hubei, People’s Republic of China; 2 Centre of Health Administration and Development Studies, Hubei University of Medicine, Shiyan, Hubei, People’s Republic of China; 3 Norwich Medical School, Faculty of Medicine and Health Science, University of East Anglia, Norwich, Norfolk, UK; 4 UKCRC Centre for Diet and Activity Research (CEDAR), Cambridge, UK

**Keywords:** Hypertension, Quality of hypertension care, Community-based interventions, Primary care, Low and middle income countries, Systematic review

## Abstract

**Background:**

Hypertension is a serious public health problem in China and in other developing countries. Our aim is to conduct a systematic review of studies on the effectiveness of community interventions for hypertension management in China.

**Methods:**

China National Knowledge Infrastructure, PubMed, and references of retrieved articles were searched to identify randomised or quasi-randomised controlled studies that evaluated community hypertension care in mainland China. One reviewer extracted and a second reviewer checked data from the included studies.

**Results:**

We included 94 studies, 93 of which were in Chinese language, that evaluated the following interventions: health education, improved monitoring, family-support, self-management, healthcare management changes and training of providers. The study quality was generally poor, with high risk of biased outcome reporting and significant heterogeneity between studies. When reported, the vast majority of the included studies reported statistically significantly improved outcomes in the intervention group. By assuming zero treatment effects for missing outcomes, the weighted reduction in the intervention group was 6∙9 (95% CI: 4∙9 to 8∙9) mm Hg for systolic BP, and 3∙8 (95% CI: 2∙6 to 5∙0) mm Hg for diastolic BP. Exploratory subgroup analyses found no significant differences between different interventions.

**Conclusions:**

After taking account of possible reporting biases, a wide range of community interventions for hypertension care remain effective. The findings have implications for China and other low and middle income countries facing similar challenges. Because of significant heterogeneity and high risk of bias in the available studies, further well designed studies should be conducted in China to provide high quality evidence to inform policy decisions on hypertension control.

## Background

Elevated blood pressure is causally associated with the risk of cardiovascular disease (CVD) [1]. While average blood pressure (BP) appears to be on the decline in high income countries, it is rising fast in many low and middle income countries [2]. The overall prevalence of hypertension in Chinese adults was 13∙6% in 1991 [3], and it increased to 18∙1% in 2004 [4]. According to a survey in 2004 in China, only 31% of patients with hypertension were aware of the condition, 23% received antihypertensive medications, and only 8% achieved satisfactory control of hypertension [5]. A more recent study in Chinese urban areas found that the proportion of hypertensive patients who received treatment was only 28%, and adequate BP control was achieved in only 3.7% [6]. The rate of hypertension control in China is low compared to high income countries [7].

As a consequence of inadequate access to and poor quality of health care, the age- and sex- standardised mortality rate from stroke in 2004 in China was more than three times as high as in Japan and other high income countries [8]. In China, 60% of deaths from CVD were attributable to high blood pressure [9]. It has been recommended that adequate control of hypertension in developing countries could be achievable by community based programmes and by upgrading primary healthcare systems [10]. An important barrier to scaling up the efforts at combating non-communicable diseases in low and middle income countries has arguably been the scarcity of evidence of (affordable) effective interventions that have been proven to work in the specific limited resource country context [11,12].

Existing systematic reviews on interventions for hypertension care include mainly studies conducted in high income countries, typically published in the English language literature [13,14]. This systematic review aims at evaluating the effectiveness of community interventions for the management of patients with hypertension in China, undertaking a deliberate effort to exploit the potentially significant evidence base published in the Chinese language. The relevance of this research is to be seen within the broader context of current efforts to tackle the substantial burden of non-communicable diseases in China[15] as well as globally [12,16].

## Methods

### Data sources and searches

We searched the China National Knowledge Infrastructure (CNKI) in September 2010, using the following terms: ‘community’, ‘intervention’, ‘control’, and ‘hypertension’. In addition, we searched PubMed in April 2011, to identify relevant studies published in Chinese or English languages. We also checked references of retrieved articles to identify further relevant studies. The search strategies used is shown in Additional file 1.

Identification of relevant studies was carried out by one reviewer (FS) and checked by a second reviewer (ZXL, YC or YL). Titles and abstracts yielded by searching bibliographic databases were first examined. Then the full publications of possibly relevant studies based on titles and abstracts were retrieved and examined for inclusion or exclusion.

### Study selection

We included studies that met the following criteria:

· Eligible participants were those diagnosed with hypertension in a specified geographically defined community (for example, cities, districts, counties, villages, or work sites).

· Interventions investigated were provided by primary care workers including general practitioners, village doctors, or nurses, to improve the management of hypertension in the community. Eligible interventions include education directed toward patients, training of care providers, organisational changes, frequent monitoring, patient self-management, and family support.

· Randomised or quasi-randomised controlled studies conducted in mainland China. Quasi-random methods of allocation included alternation, date of birth, or patient record code [17].

To assess the risk of possible outcome reporting bias, we included studies that met the above inclusion criteria but did not provide sufficient data on relevant outcomes [18]. We excluded studies according to the following exclusion criteria: studies conducted in regions outside mainland China, trials that compared different pharmacological interventions, and non-randomised or before-after studies. Studies were also excluded because of suspected plagiarism.

### Data extraction and quality assessment

We used a data extraction sheet (Additional file 2) to extract the following data from the included studies: study characteristics, interventions investigated, and study results. The quality of included studies was assessed using a checklist based on “Quality Assessment tool for Quantitative Studies” (Additional file 3). Data extraction and quality assessment were conducted by one reviewer (FS) and checked by a second reviewer (ZXL or SYC).

### Data synthesis and analysis

The difference in blood pressure between groups is considered as the primary outcome. The secondary outcomes were the proportion of patients on anti-hypertension treatment, and the proportion of patients with adequate control of blood pressure. We adopted the definitions used by individual studies for anti-hypertension treatment and adequate control of blood pressure.

The effect estimates were mean difference (MD) for continuous data and odds ratio (OR) for binary data. Heterogeneity across studies was tested using the *χ*^*2*^ test and quantified by *I*^*2*^ statistic [17]. We used DerSimonian-Laird method to conducted random-effects meta-analyses, in which individual studies were weighted by the inverse of the sum of within-study variance plus between-study variance [19]. The effective sample sizes of cluster randomised studies were estimated based on an assumed intra-cluster correlation (ICC) of 0.04 [17,20].

Statistical analyses were performed using STATA^@^ software. Exploratory subgroup analyses were conducted according to types of interventions, participant characteristics, study design and quality. Differences between subgroups were tested using random-effects meta-regression. We investigated the risk of publication bias by using funnel plots [21]. To test funnel plot asymmetry, Egger’s method was used for continuous outcomes [22], and Peters’ method for binary outcomes [23]. Sensitivity analyses were conducted to adjust outcome reporting bias by assuming zero treatment effects for all missing outcomes. The reporting of this meta-analysis was in line with the Preferred Reporting Items for Systematic Reviews and Meta-analyses: the PRISMA statement [24].

## Results

**Figure 1:**
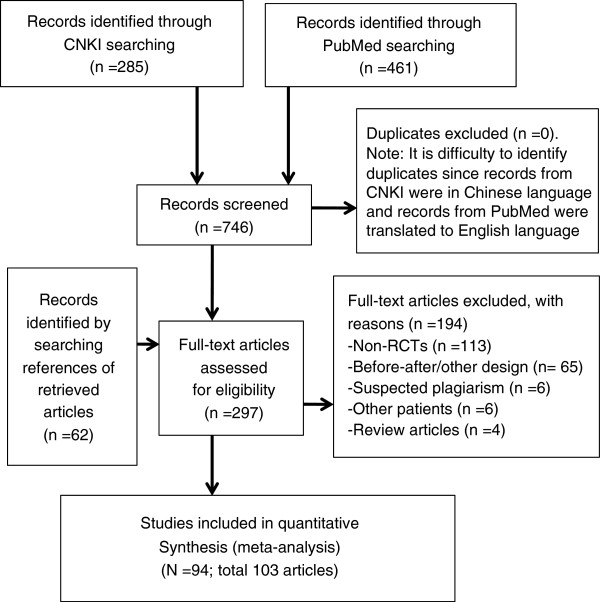
Study identification flow diagram.

The process of study identification is shown in Figure 1. We included 94 randomised controlled studies (see Additional file 4 for references of these studies). The main reason for exclusion was the non-randomised (n = 113) or before-after design (n = 65).

The main characteristics of the included studies are summarised in Additional file 5. Of the 94 included studies, 93 were published in Chinese language and only one in English. Education of patients was the most commonly evaluated intervention in the included studies. Other interventions investigated were increased monitoring of blood pressure, family support, patient self-management, organisational changes, and training of care providers (Table 1). The main care providers were community nurses and general practitioners. Community interventions were delivered in community hospitals, community centres, or at patients’ home.

**Table 1:** Interventions evaluated and care providers – hypertension care in the community in China

	**General practitioners**	**Community nurses**	**GPs & nurses**	**Unclear providers**	**Total**
**Education of patients**	28	36	6	16	86
**Increased BP monitoring**	20	22	2	2	46
**Improved family support**	4	7	1	1	13
**Facilitating self-management**	2	4	1	2	9
**Organisational changes**	4	1	2	1	8
**Training of care providers**	1	1	1	1	4
**Total**	30	42	6	16	94

**Note to Table 1**: A study may have evaluated more than one intervention.

Patients in 70 studies were recruited from the community or not specified, while patients in 24 studies were explicitly recruited from outpatients or discharged inpatients in community hospitals. In most of the included studies, there were no specific patient inclusion or exclusion criteria additional to the diagnosis of hypertension. Six studies included only old hypertensive patients (≥ 60 year of age), while two studies included only younger patients (≤35 or ≤55 years of age). Four studies included patients with newly diagnosed hypertension, one included patients with uncontrolled hypertension, and one included patients with pre-hypertension.

### Quality of included studies

Most of the included studies did not describe randomisation methods, although all stated that patients were randomly allocated between groups (Table 2). Randomisation sequence generation was clearly adequate in only three studies. There are nine quasi-randomised studies in which patient allocation was according to their medical record numbers. Allocation concealment was unclear in all of the included studies. Only five studies explicitly used blinding, although none provided details on methods.

**Table 2:** Results of quality assessment of included studies

**Quality items**	**No. of studies**
Randomisation sequence generation:	
- Adequate	3
- Inadequate (quasi-RCT)	9
- Unclear	76
- Cluster design	6
Allocation concealment:	
- Unclear	94
Blinding (any):	
- Yes	5
- No	89
Sample representativeness:	
- Very likely	22
- Somewhat likely	47
- Unlikely or can’t tell	25
Sample size calculated:	
- Yes	3
- Unclear or No	91
Comparability between groups:	
- Reported no significant differences	87
- Some difference or can’t tell	7
Contamination between arms:	
- Unlikely	18
- Somewhat likely	68
- Very likely	8
Loss to follow up:	
- Reported	22
- No reported	72
Outcomes reported:	
- Blood pressure	48
- Hypertension treatment	54
- Adequate hypertension control	56

Six of the included studies were cluster randomised studies. Only one of the six studies considered intra-class correlation in the calculation of sample size and outcome analyses [20]. The other five cluster randomised studies designed the study and analysed data without appropriate adjustment for clustering.

Patients in 69 studies were considered to be likely or somewhat likely to be representative of general community patients with hypertension. Sample size calculation was explicitly reported in only three studies. Most (n = 87) of the included studies stated briefly in one or two sentences that there were no statistically significant differences in certain baseline patient characteristics between groups. However, the comparability of baseline patient characteristics should be interpreted with great caution because of the small sample sizes and the unclear selection of patient characteristics. Loss to follow up was explicitly reported in only 22 studies. Contamination between the intervention and control was considered by the reviewers to be likely or somewhat likely in most studies (n = 76).

Of the 94 included studies, 48 reported blood pressure as outcome, 54 reported the number of patients on regular hypertension treatment, and 56 reported the number of patients with adequate blood pressure control. Only 14 studies reported all three outcomes, and data from three studies was insufficient for any of the relevant outcomes.

### Main pooled results

**Figure 2:**
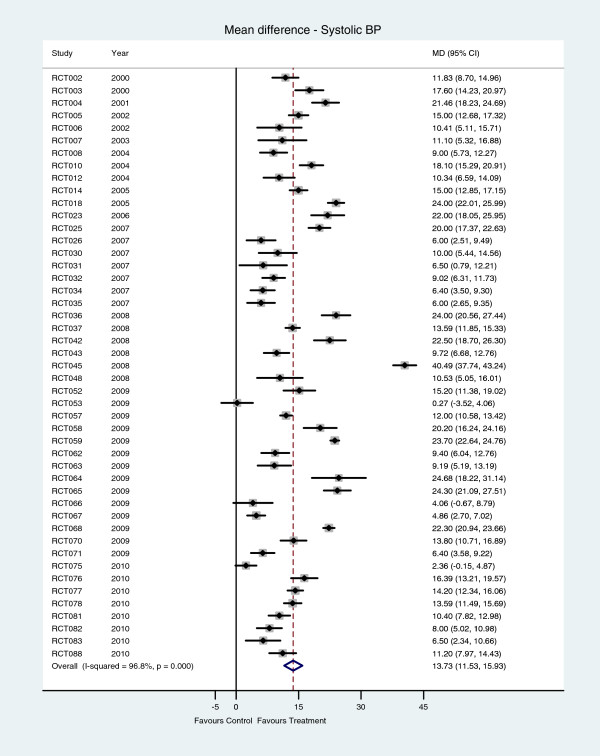
Systolic blood pressure – mean difference between hypertension care and control interventions.

According to data from 47 studies, there was statistically significant heterogeneity across studies (*I*^*2*^ = 96∙8%, P < 0∙001). With only three exceptions, systolic blood pressure was statistically significantly lower in the intervention group (Figure 2). The random-effects meta-analysis suggested that systolic blood pressure was reduced on average by 13∙73 mm Hg (95% CI 11∙53 to 15∙93) in the intervention group.

**Figure 3:**
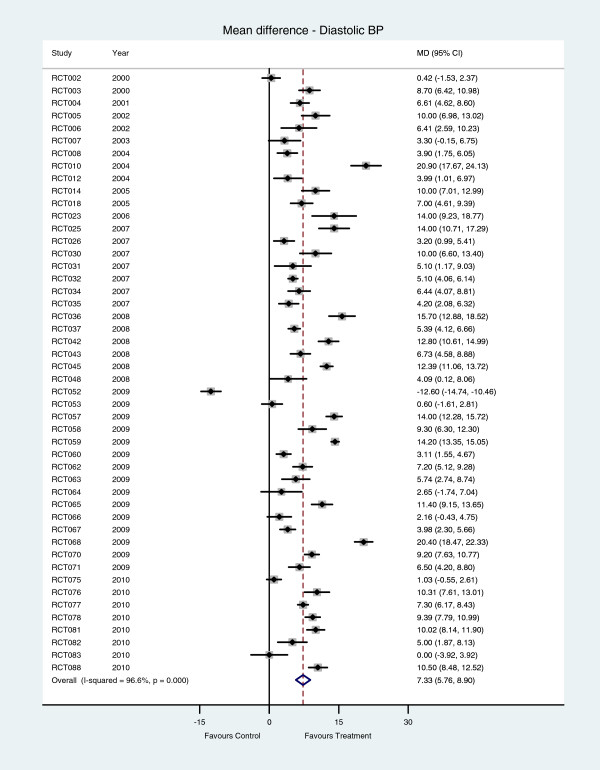
Diastolic blood pressure - mean difference between hypertension care and control interventions.

Diastolic blood pressure in the intervention group was statistically significantly lower than that in the control group in 41 of the 48 studies that reported this outcome (Figure 3). The pooled mean difference in diastolic blood pressure is 7∙33 mm Hg (95% CI 5∙76 to 8∙90), and the heterogeneity across studies was statistically significant (*I*^*2*^ =96∙6%, P < 0∙001).

**Figure 4:**
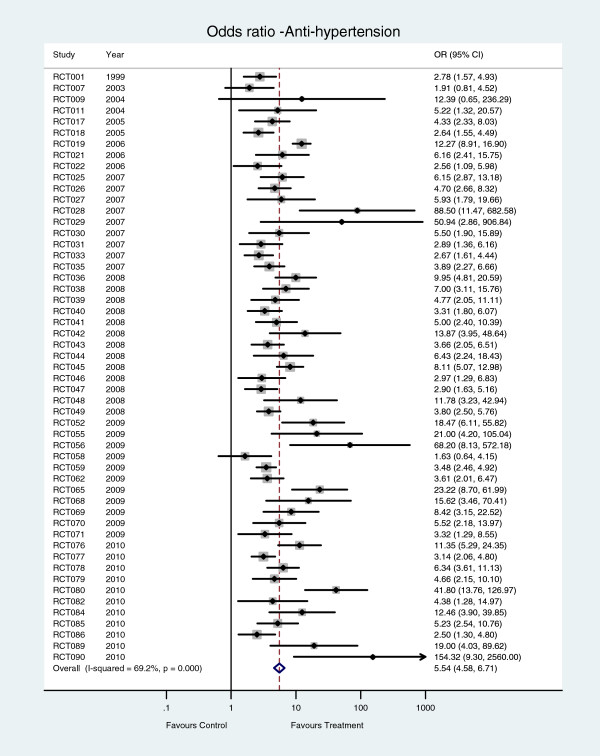
Number of patients on antihypertensive treatment – hypertension care versus control interventions.

The number of patients on antihypertensive treatment was statistically significantly increased in 51 of the 53 studies that reported this outcome (Figure 4). Heterogeneity across studies was statistically significant (*I*^*2*^ = 74∙8%, P < 0∙001). The pooled odds ratio for antihypertensive treatment was 5∙54 (95% CI 4∙58 to 6∙70).

**Figure 5:**
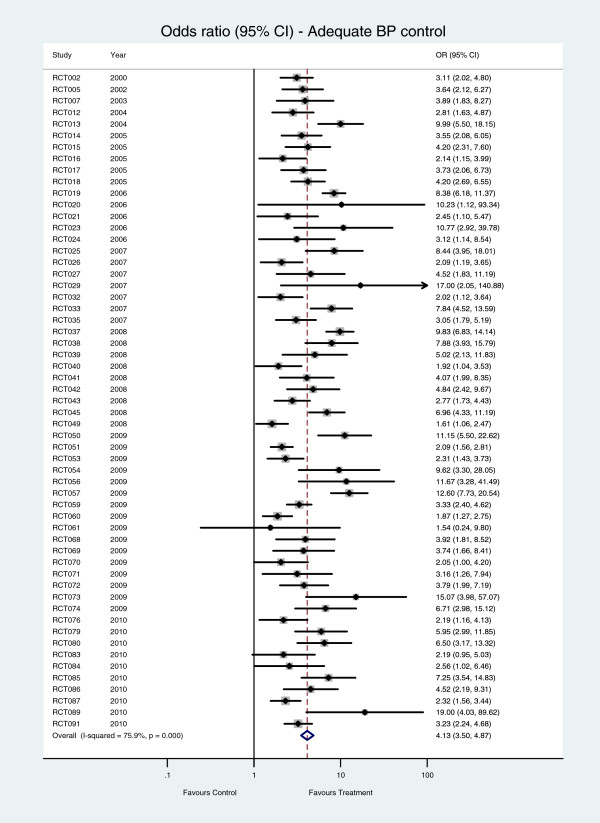
Number of patients with adequate BP control – hypertension care versus control interventions.

The proportion of patients with adequate blood pressure control in the intervention group was statistically significantly higher than that in the control group in 55 of the 57 studies (Figure 5). The pooled odds ratio for adequate control was 4∙13 (95% CI 3∙50 to 4∙87), and heterogeneity across studies was statistically significant (*I*^*2*^ = 75∙9%, P < 0∙001).

### Subgroup analyses

**Figure 6:**
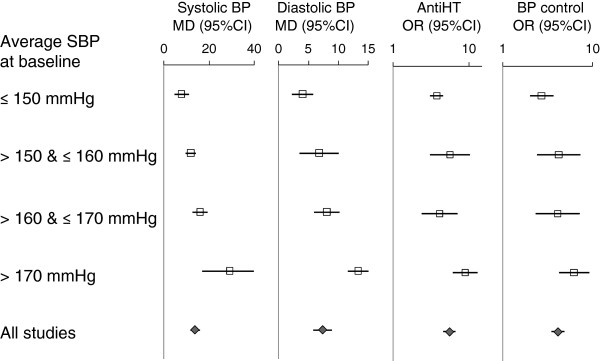
Treatment effects and the average systolic blood pressure at baseline: results of subgroup meta-analyses.

The results of exploratory subgroup analyses revealed no clear differences in treatment effects by types of interventions and care providers (Table 3). Significant heterogeneity was still present in almost all of the subgroup meta-analyses. Overall, there were no statistically significant, consistent and plausible differences in the results of meta-analyses between subgroups in terms of types of interventions, patient characteristics, care providers, study design features and quality. However, there was a consistent tendency that treatment effects were positively associated with the average systolic blood pressure at baseline (Figure 6).

**Table 3:** Results of meta-analyses by care providers and interventions (systolic BP outcome)

**Subgroups**	**No. of studies**	**Mean difference (95% CI)**	**I**^**2**^
**All studies**	47	13.7 (11.5, 15.9)	96.8%
**By care providers:**			
Community nurses only	19	14.2 (10.8, 17.7)	96.8%
General practitioners only	13	14.9 (10.0, 19.8)	97.9%
GP and nurses	5	11.2 (6.5, 15.9)	89.3%
**By interventions:**			
Education of patients	43	14.0 (11.7, 16.2)	96.7%
Training of care providers	3	11.5 (−1.8, 24.8)	99.0%
BP monitoring	23	14.8 (11.5, 18.1)	96.4%
Self-management	6	13.7 (10.4, 17.0)	89.8%
Family support	8	14.5 (9.9, 19.2)	96.0%
Organisational changes	6	12.2 (7.3, 17.1)	94.4%

**Note to Table 3:** Heterogeneity was statistically significant (P < 0.001) for all subgroups.

### Impact of outcome reporting bias

**Figure 7:**
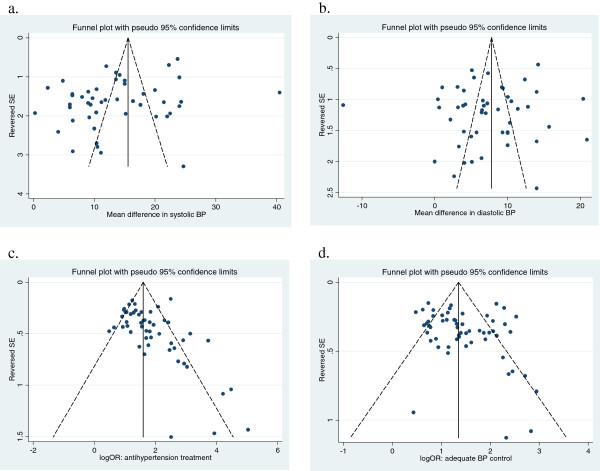
Funnel plots of the main meta-analyses – hypertension care versus control interventions.

Figure 7 shows the funnel plots for the meta-analyses. The funnel plot for mean differences in systolic BP indicates that smaller studies are associated with the smaller treatment effect (Egger’s test P = 0∙02). The funnel plots are not statistically significantly asymmetric for the other three outcomes (Figure 7).

The risk of outcome reporting bias is considerable, since data on the relevant outcomes was not available from 40% to 49% of the included studies, and it is likely that studies did not report outcomes because of non-significant or negative results. We conducted sensitivity analyses by imputing zero treatment effects (with average standard deviations) in studies that did not report a relevant outcome. As compared with meta-analyses using reported data only (MD 13.7 mm Hg, 95% CI: 11.5 to 15.9), the pooled mean difference in systolic BP in meta-analyses using both reported and imputed data is much reduced but do remain statistically significant (MD 6.9 mm Hg, 95% CI: 4.9 to 8.9).

## Discussion

We included studies that evaluate different types of interventions, including health education directed to patients, increased monitoring, family support, patient self-management, training of care providers, and care system changes. There are considerable overlaps between different categories, and interventions with multiple components were common. Health education, the most commonly evaluated intervention, was usually a necessary component for patient self-management, family support, and improved BP monitoring.

Based on data from 94 studies in China (with 21,942 patients in total), these interventions for the hypertension care in the community were highly effective. However, the findings need to be interpreted with great caution because of statistical heterogeneity, the poor quality of the included studies, and the risk of biases.

Interventions for hypertension management have been evaluated in existing systematic reviews that included studies mainly conducted in developed countries [13,14]. In a systematic review of 57 studies, Walsh et al. found that several quality improvement strategies were associated with improved hypertension care [14]. A Cochrane systematic review of 72 randomised controlled studies concluded that self-monitoring and vigorous stepped care approach could improve hypertension control, while educational interventions were unlikely to be effective [13].

Findings from studies conducted in developed countries may not be generalisable to developing countries. The quality of current hypertension care in developing countries is generally poorer than that in developed countries, so the potential for improvement is different between countries or regions. This may be one of several possible reasons that studies on hypertension interventions in China tended to report greater effects than studies in developed countries. That is, there may have been more room for improvement in China. Similarly, a study in Pakistan reported that home health education and special training of general practitioners significantly improved hypertension care and clinical outcomes [25,26].

The recently updated Cochrane review [13] had no language restriction, but included none of the 94 studies included in the present review. This is due to the fact that only a limited number of relevant journals in Chinese language are indexed in PubMed and other international databases. Only two of the 94 included studies were identified by searching PubMed in this systematic review – a finding that by itself has important implications for future attempts to tap the evidence base from the potentially non-negligible (or even substantial) research published in non-English domestic journals. We also identified two relevant meta-analyses published in Chinese language. One meta-analysis in 2000 included 12 quasi-experimental studies on interventions at the population level (9 in high income countries and 3 in China) [27]. Another meta-analysis focused on community nursing care of elder patients with hypertension, and included six studies in China [28]. Both meta-analyses concluded that community-based interventions are effective in the management of hypertension.

Because of significant heterogeneity in results across studies, pooling of results in meta-analysis may be controversial. Reduced within-study variance (as a consequence of more and/or larger studies) is associated with increased heterogeneity statistic *I*^*2*^, given the same between-study variance [29,30]. For this reason, the clinical relevance of the observed heterogeneity, not the statistical significance, should be the main concern about whether the results could be pooled. In the current systematic review, statistically heterogeneous results from individual studies were of the same or similar clinical interpretations, consistently indicating the positive effects of health care interventions in the community. Therefore, we conducted pooled analyses in this systematic review, despite statistically significant heterogeneity.

We conducted subgroup analyses to explore possible causes of statistical heterogeneity across studies. The only consistent finding was the association between baseline blood pressure and treatment effects, suggesting that hypertension management may be more effective in higher risk patients. There were no consistent subgroup differences between other patient characteristics, interventions, and primary care providers. It may be interesting to note that interventions provided by community nurses were as effective as those by general practitioners (Table 3).

The quality of the included studies was generally poor, confirming findings from previous studies [31]. There is a lack of details on baseline patient comparability in most studies, although it was often claimed that there were no statistically significant differences in certain baseline variables between groups. A previous study found that many claimed randomised studies published in Chinese journals may not really be randomised [32]. Treatment effects of hypertension care interventions may have been exaggerated due to the poor study quality in published Chinese studies.

As a consequence of outcome reporting bias, statistically non-significant outcomes are less likely to be reported than significant outcomes in published reports [18]. We suspected the existence of outcome reporting bias in studies included in this review. Although only a few studies estimated statistical power in the design, the vast majority of reported outcomes were statistically significant. In addition, data on the relevant outcomes was available from only 51% to 60% of the included studies. It is likely that a missing outcome was measured but not reported in some studies. Therefore, we conducted sensitivity analyses to estimate possible impact of reporting bias on the effect estimates, by assuming zero differences between groups for missing outcomes. The estimated effect sizes using both reported and imputed data were reduced by 38% to 48%, as compared to that using only reported data. According to sensitivity analyses including imputed data, community-based interventions for hypertension care reduced systolic BP by 6.9 mm Hg.

Findings of this systematic review have very important public health implications. The total number of cardiovascular deaths (CVD) related to blood pressure in China was estimated to be about 2.3 million in 2005 [9]. Research evidence suggested that lowering systolic blood pressure by 7 mm Hg reduces the risk of CVD events on average by about 20% to 25% [33]. Therefore, a conservative estimate is that community interventions for hypertension care could avoid 500, 000 cardiovascular deaths each year in China, plus a huge number of prevented non-fatal CVD events. These highly effective community interventions do not depend on expensive technologies or highly trained specialists. Therefore, these interventions are very appropriate in China and (most likely) in many other low and middle income countries. However, public policy measures are required to establish, improve and upgrade community health services to cope with the increasing burden of chronic diseases.

## Conclusions

After taking account of the potential bias we find that community interventions provided by primary care professionals in China remain effective for managing patients with hypertension. Hence, policymakers in China interested in reducing the burden of non-communicable diseases should consider very seriously the expected benefits that community interventions to reduce hypertension could bring. The findings likely also bear useful messages for other similarly resource-constrained low and middle income countries. Because of significant heterogeneity and high risk of bias in the available studies, further well designed large scale studies should be conducted in China to provide high quality evidence to inform policy-decisions on hypertension control.

## Abbreviations

BP, Blood pressure; CNKI, China National Knowledge Infrastructure; CVD, Cardiovascular disease; GP, General practitioner; ICC, Intra-cluster correlation; MD, Mean difference; OR, Odds ratio; RCT, Randomised controlled trial.

## Competing interests

We declare that we have no conflict of interest. No specific funding received for this systematic review.

## Authors’ contributors

FS and ZXL conceived the idea and prepared a draft review protocol. YC and FS conducted literature search. YC, SYC and YL collected identified articles. FS extracted data from the included studies. SYC and ZXL checked extracted data. FS analysed data and prepared manuscript. ZXL, SYC, YC, LY, MB and MS helped results interpretation and critically commented on and revised the manuscript. Fujian Song had full access to all the data in the study and takes responsibility for the integrity of the data and the accuracy of the data analysis. All authors read and approved the final manuscript.

## Pre-publication history

The pre-publication history for this paper can be accessed here:

http://www.biomedcentral.com/1472-6963/12/216/prepub

## Supplementary Material

Additional file 1 Literature search strategy. Click here for file

Additional file 2 Sheet used to extract data from controlled studies on hypertension care in China. Click here for file

Additional file 3 Method used to assess the quality of included studies. Click here for file

Additional file 4 References of included studies – hypertension care in the community in China. Click here for file

Additional file 5 Main characteristics of included studies –hypertension care in the community in China. Click here for file
