# Supplementary material for: Effectiveness of interventions for hypertension care in the community – a meta-analysis of controlled studies in China
Source: BMC Health Serv Res. 2012 Jul 24;12:216. doi: 10.1186/1472-6963-12-216 (PMC3416668; doi:10.1186/1472-6963-12-216)
Supplement: Additional file 1 — Literature search strategy. [file 1472-6963-12-216-S1.pdf]

# Additional File 1: Literature search strategy – hypertension care in the community in China

1. China National Knowledge Infrastructure (CNKI) were searched, using the following key words:

> **“社区” AND “干预” AND “对照” AND “高血压”** (translation: community AND intervention AND control AND hypertension).

2. PubMed was searched using the following terms (limited to title/abstract):

> ("hypertension" OR "blood pressure" OR "cardiovascular" OR "stroke" OR "cerebrovascular") AND ("community"[All Fields] OR "population"[All Fields] OR "primary care"[All Fields] OR "comprehensive"[All Fields]) AND ("intervention"[All Fields] OR "health education"[All Fields] OR "management"[All Fields] OR "control"[All Fields] OR "prevention"[All Fields]) AND ("China"[All Fields] OR "Chinese"[All Fields]) AND (Humans[Mesh] AND (Clinical Trial[ptyp] OR Meta-Analysis[ptyp] OR Randomized Controlled Trial[ptyp] OR Comparative Study[ptyp] OR Controlled Clinical Trial[ptyp] OR "cohort"[All Fields] OR "evaluation"[All Fields]))

3. References of retrieved articles will also be examined to identify relevant studies.
