# Supplementary material for: Effectiveness of interventions for hypertension care in the community – a meta-analysis of controlled studies in China
Source: BMC Health Serv Res. 2012 Jul 24;12:216. doi: 10.1186/1472-6963-12-216 (PMC3416668; doi:10.1186/1472-6963-12-216)
Supplement: Additional file 2 — Sheet used to extract data from controlled studies on hypertension care in China. [file 1472-6963-12-216-S2.pdf]

Additional File 2: Sheet used to extract data from controlled studies on hypertension care in China

Study characteristics

Code	Study	Year	Design	Year of study	Size	Follow up year	Setting	Region	Participants	Age	Male (%)

Intervention, description:

Main components						Providers				
Patient education	Provider education	Improved monitor	self-manage	Family support	Organiza-tional	Unclear	GP/ Doctor	Nurse	Other	Control

Results

Systolic BP (mean+/-SD) mmHg

Baseline Intervention			Baseline Control			Endpoint Intervention			Endpoint Control		
n	Mean	SD	n	Mean	SD	n	Mean	SD	n	Mean	SD

Diastolic BP (mean+/-SD) mmHg

Baseline Intervention			Baseline Control			Endpoint Intervention			Endpoint Control		
n	Mean	SD	n	Mean	SD	n	Mean	SD	n	Mean	SD

Regular treatment rate

Baseline Intervention			Baseline Control			Endpoint Intervention			Endpoint Control		
n	event	%	n	event	%	n	event	%	n	event	%

HBP Control rate

Baseline Intervention			Baseline Control			Endpoint Intervention			Endpoint Control		
n	event	%	n	event	%	n	event	%	n	event	%

eBox 3. Method used to assess the quality of included studies

"Quality Assessment tool for Quantitative Studies" (http://www.nccmt.ca/registry/view/eng/14.html) was developed by the Effective Public Health Practice Project (EPHPP) to the assessment of studies on public health interventions. We simplified this tool and used the following checklist to assess the quality of included studies in this review:

- Study design: RCT, CT, quasi-experimental
- Participant representativeness: Are study participants likely to be representative of the target population? (very likely; somewhat likely; not likely; can't tell)
- Comparability between groups within a study: Were there between group differences for important confounders reported in the paper? (Yes, No, Can't tell)
- Blinding: Was the outcome assessor blinded to the intervention status? (Yes, No, Not reported, Not applicable)
- Withdrawal/drop outs: rate of withdrawals /drop-outs during study period; or Not Reported, NA.
- Sample size: Sample size or power calculation? (Yes, Partially, No)
- Intervention contamination: Control participants also received some interventions? (Very likely, somewhat likely, unlikely; not applicable)
- Completeness of outcomes measured/reported: BP outcome (yes/no), Treatment compliance outcome (Yes/no), Hypertension control outcome (Yes/no), CVD outcome (yes/no)
