# Supplementary material for: Effectiveness of interventions for hypertension care in the community – a meta-analysis of controlled studies in China
Source: BMC Health Serv Res. 2012 Jul 24;12:216. doi: 10.1186/1472-6963-12-216 (PMC3416668; doi:10.1186/1472-6963-12-216)
Supplement: Additional file 4 — References of included studies – hypertension care in the community in China. [file 1472-6963-12-216-S4.pdf]

Additional File 4: References of included studies –hypertension care in the community in China

Code	References (English translated)	Original references
RCT001	Liu JP, He XL, Reng XD. Study of Health Education to Raise Treatment Compliance in Patients with Primary Hypertension. Journal of PracticaI Nursing1999,15(9):58-60	刘建萍, 何晓俐, 任晓丹.健康教育对提高原发性高血压患者治疗依从性的研究[J].实用护理杂志, 1999, 15(9): 58.
RCT002	Wang YS, Zhong HS, Kuang XC, Xiao DM, Mao J, et al. Study of Community Rehabilitation Intervention to the Elderly with Borderline hypertension. Morden Rehabilitation 2000, 4(12): 1823-24.	王育珊 钟华荪 邝星驰 肖冬梅 毛俊. 老年临界高血压患者的社区康复干预. 现代康复. 2000 年 第 12 期
RCT003	Xiao DM, Zhong HS, Kuang XC, Wang YS, Feng MH. Study on Community Intervention to the Drug Administration Behavior for Gerontal Hypertensive Patients. Morden Rehabilitation 2000, 4(11): 16-17	肖冬梅 钟华荪 邝星弛 王育珊 冯美欢. 老年高血压病人服药行为的社区干预. 现代康复. 2000 年 第 13 期
RCT004	Wu X, Xu B. Community Health Education for Hypertensive Patients. Journal of Nursing Science 2001,16(7) : 438-440.	吴熹 许兵. 高血压病病人的社区健康教育. 护理学杂志. 2001 年 第 07 期
RCT005	Wang SS. A Community-based Prospective Control Study of Comprehensive Intervention in Hypertensive Patients. Chin Jof Int Med 2002,41(3) : 168-171.	王顺铨. 对社区高血压病患者进行综合干预的前瞻性对照研究. 中华内科杂志. 2002 年 第 03 期
RCT006	Chen M. Study of Comprehensive Nursing Intervention with Hypertensive Patients in a Community. Chinese General Practice 2002, 5(11) : 911-912.	陈梅. 社区高血压患者综合护理干预研究. 中国全科医学. 2002 年 第 11 期
RCT007 (PubMed)	Yu PL, Ye W, Liu XR, Liu YJ, Zhang J, Bai XL, et al. Evaluation on the effectiveness for self-management of hypertensive patients in a community. Zhonghua Liu Xing Bing Xue Za Zhi 2003;24(9):790-3.	于普林 叶文 刘雪荣 刘耀军 张静 白筱玲. 社区高血压患者自我管理的效果评估. 中华流行病学杂志 2003 年 第 09 期
RCT008	Huang ZQ, Chen WY, Huang Y, Luo S. Study of intervention to aged patients with hypertension in Buji community of Shenzhen. Clinical Education of General Practice 2004,2(4) : 232-233.	黄智前 陈伟业 黄吟 罗山. 深圳市布吉社区老年人高血压病患者的干预研究. 全科医学临床与教育 2004 年 第 04 期

RCT009	Xiao CL. Study on the influence of Community nursing intervention to treatment compliance of hypertensive patients. Medical Journal of Qilu 2004,19(5) : 429-430.	肖翠林. 社区护理干预对高血压病人服药依从性的影响. 齐鲁医学杂志 2004 年 第 05 期
RCT010	Wang XF, Wang JP, Ouyang RC, Liao YL, Lv ZQ, Ma HB. A random control study of community intervention with hypertensive patients. Chinese Journal of Primary Medicine and Pharmacy 2004,11(10) : 1169-1170.	王熙福 王坚平 欧阳荣超 廖玉联 吕仲群 马红宝. 高血压患者社区干预对照研究. 中国基层医药. 2004 年 第 10 期
RCT010a	Ma HB. Discuss on the model of nursing intervention to hypertensive patients in a community. Modern hospital 2005,5(6) : 89-90.	马红宝. 社区高血压病患者护理干预模式探讨. 现代医院 2005 年 第 06 期
RCT011	.Zhang YL, Cai LP, Yang XQ, Kong Q, Li JM, Meng QY. Study on nursing intervention with hypertensive patients in a Community. Modern nursing 2004,10(3) : 276-277.	张雅雯 蔡兰平 杨晓秋 孔曲 李金梅 孟庆义. 社区高血压病患者的护理干预. 现代护理 2004 年 第 03 期
RCT012	Fu DP, Yang KJ, Wang JL, Sun W, Teng XQ, Ding YM. Effectiveness of community-based hypertension self-management on health status and blood pressure control. Chin J Hea Edu 2004,20 (10) : 869-871.	傅东波 杨柯君 王敬丽 孙玮 滕秀琴 丁永明. 社区高血压自我管理模式及血压控制效果分析. 中国健康教育 2004 年 第 10 期
RCT012a	Fu DP, Ding YM, Yang KM, Sun W, Wang JL, Fu H. Evaluation of hypertension self-management patient education program. Fudan University Journal of Medical Science 2005,32(3) : 284-289.	傅东波 丁永明 杨柯君 孙玮 王敬丽 傅华. 高血压自我管理健康教育项目效果评价. 复旦学报(医学版) 2005 年 第 03 期
RCT012b	Yang KJ, Wang JL, Sun W, Ding YM, Fu DP, Fu H. Analysis on effectiveness of hypertension self-management model and hypertension classification management model on blood pressure control. Shanghai Journal of Preventive Medicine 2005,17(8) : 368-370.	杨柯君 王敬丽 孙玮 丁永明 傅东波 傅华. 高血压自我管理模式与分级管理模式效果比较. 上海预防医学杂志 2005 年 第 08 期
RCT012c	Wang JL, Yang KJ, Fu DP, Ding YM, Fu H. Eveluation of the effectiveness of hypertension self-management program. Chinese General Practice 2006,9(1) : 35-36.	王敬丽 杨柯君 傅东波 丁永明 傅华. 高血压自我管理项目对自我效能的效果评价. 中国全科医学 2006 年 第 01 期
RCT013	Wang SF. 205 cases of hypertension prevention and control in a community. Zhejiang Clinical Medicine 2004,6(8):717-718.	王淑芬. 205 例高血压病的社区防治[J]. 浙江临床医学, 2004, 6(8) : 717

RCT014	Zhang XH. Study on the influence of community comprehensive intervention to hypertension treatment. Journal of Shanxi Medical College for Continuing Education 2005,15(2) : 22-23.	张晓华. 社区综合干预对高血压病治疗的影响. 山西职工医学院学报 2005 年 第 02 期
RCT015	Zhu Y. A double-blind randomized controlled trial on community intervention to treat primary hypertension. Modern Journal of Integrated Traditional Chinese and Western Medicine 2005,14(7) : 869.	朱颖. 社区干预治疗原发性高血压的双盲随机对照实验. 现代中西医结合杂志. 2005 年 第 07 期
RCT016	Zhu J, Wang P, Wu J. A survey on the blood pressure control situation of community nursing intervention to hypertensive patients. Journal of Branch Camp First Military Medical University 2005,28(1) : 81-83.	朱健 汪萍 吴君. 社区护理干预对高血压患者血压控制情况调查. 第一军医大学分校学报. 2005 年 第 01 期
RCT017	Yang YF,Hong Q, Chu WG, Tang J, Zhang L ,Cheng LM. Evaluation of raising the treatment compliance on the family's integrated intervention. Chinese Primary Health Care 2005,19(4) : 43-44.	杨运方 洪倩 储文革 汤静 张丽 程利民. 家庭综合干预是改善高血压患者治疗依从性的有效措施. 中国初级卫生保健 2005 年 第 04 期
RCT018	Sun HH, Zhou H, Chen SD, Li BJ, Zhang X. Influence of Health education to the aged hypertensive patients in a community. Journal of Guangdong College of Pharmacy 2005, 21(5) : 598-599.	孙宏慧 周和 陈思东 李伯佳 张信. 健康教育对社区老年高血压病人的影响. 广东药学院学报 2005 年 第 05 期
RCT019	Zhao XL, Ye DJ, Yang JG, Zhang DK, Shen HB. Evaluation of the community based intervention with NAH program on the population with hypertension. Journal of Disease Control and Prevention 2006,10(5) : 482-484.	赵小兰 叶东进 杨建国 张德坤 沈洪兵. NAH 系统治疗管理方案对高血压人群社区干预效果评价. 疾病控制杂志 2006 年 第 05 期
RCT020	Du ZH, Wu WG, Zhou JD. Observation of general practitioner team intervention of primary hypertensive patient: an analysis of 40 cases.Chinese General Practice 2006, 13(4): 892-893.	杜兆辉 吴卫国 周建东. 40 例原发性高血压患者全科团队服务式干预的效果观察和思考. 中国全科医学 2006 年 第 23 期
RCT021	Dai KW. Nursing intervention of hypertensive patients with drug therapy in the community. Chinese Journal of Clinical Healthcare 2006,9(1) : 46-47.	戴魁梧. 社区高血压患者用药的卫生宣教. 中国临床保健杂志. 2006 年 第 01 期
RCT022	ZhuY. Discussion on the effectiveness of community intervention to with hypertensive patients in treatment compliance. Journal of Clinical and Experimental Medicine 2006, 5(12) : 2042-2043..	朱英. 对社区高血压病人治疗依从性干预作用探讨. 临床和实验医学杂志 2006 年 第 12 期

RCT023	Li LS. Study on community nursing intervention model of aged people with hypertension. Practical Preventive Medicine 2006, 13(4): 892-893.	李丽莎．老年高血压病社区护理干预效果分析[J]．实用预防医学，2006，13 (4)：892—893．
RCT024	Wu BH,Ll SM,Shi CL. Effect evaluation on health education in hypertension management in a Community. Chinese Journal of Health Education 2006, 22(6): 457-458.	吴宝恒,李淑民,石长领. 社区高血压管理中的健康教育效果评价[J]. 中国健康教育, 2006, 22:457 - 458.
RCT025	Cai LY, Su YQ, Zhao LP. Effect evaluation on self-management of elderly hypertensive patients in a community. Hebei Medical Journal 2007,29(6) : 584-586.	蔡兰英 苏月巧 赵连平．社区老年高血压患者自我管理效果评价. 河北医药 2007 年 第 06 期
RCT026	Xie XY. The role of raising treatment compliance and curative effectiveness of Community intervention to hypertension. Chinese Journal of Primary Medicine and Pharmacy 2007,14(7) : 1176-1177.	谢馨予．社区干预提高高血压治疗依从性和疗效的作用．中国基层医药 2007 年 第 07 期
RCT027	Peng ZL. Analysis of the effectiveness of Community intervention to hypertension prevention and cure. Sichuan Medical Journal 2007,28 (8) : 859-860.	彭正禄．社区干预对高血压病防治效果分析．四川医学 2007 年 第 08 期
RCT028	Huang ZQ. Influence of Community nursing intervention to hypertensive patients in treatment compliance. Today Nurse (comprehensive edition) 2007, (2) : 31,38.	黄志群．社区护理干预对高血压患者遵医行为的影响．当代护士(综合版) 2007 年 第 02 期
RCT029	Ying HX. Influence of Community nursing intervention to the patients with hypertension. Modern Journal of Integrated Traditional Chinese and Western Medicine 2007, 16(17) : 439-443.	应海霞．社区护理干预对高血压患者的影响．现代中西医结合杂志 2007 年 第 17 期
RCT030	Wu GY, Liu GH. Influence of community nursing intervention on self-management for middle-aged and aged patients with hypertension. Today Nurse (specialized edition) 2007, (7) : 6-7.	吴国英 刘桂花．社区护理干预对中年高血压患者自我管理的影响. 当代护士(学术版) 2007 年 第 07 期
RCT031	Yu HM. Influence of Community health education to the primary hypertensive patients to compliance with the doctors' orders. Journal of Qilu Nursing 2007,13(9) : 33-34.	余红梅. 社区健康教育对原发性高血压患者医嘱依从性的影响．齐鲁护理杂志 2007 年 第 09 期

RCT032	Hu QY. Influence of Family intervention to hypertensive patients. Nursing Practice and Research 2007, 4(3) : 30-31.	胡琼英. 家庭干预对高血压患者血压的影响. 护理实践与研究 2007 年 第 03 期
RCT033	Wang DY. Influence of Health education on elderly patients with hypertension in rural community. Chinese Nursing Research 2007, 21(4A)：893-895．	王冬叶．健康教育对农村社区老年高血压病病人的影响[J]．护理研究，2007，21(4A)：893—895．
RCT034	Zhang BX, Liu JZ, Wang L. Comparison on the effectiveness of nursing intervention to the Older hypertensive patients in communities. Basic Medical BBS 2007,11(10B)：919-920．	张宝香，刘景芝，王丽，等．社区老年高血压护理干预效果比较 EJ]．基层医学论坛，2007，11(10)：91—92．
RCT035	Wang CM. Effectiveness of community nursing intervention in treatment of patients with hypertension. Nursing Research. 2007,21(12C): 3377．	王彩梅．社区护理干预在高血压治疗中的作用[J]．护理研究，2007，21(12C)：3377．
RCT036	Cheng YL, Li YL, Zhong SL, Sun YF, Xue T. Effect of IKAP pattern nursing intervention on the improvement of self-care of aged patient with hypedension in community. Journal of Qilu Nursing 2008,14(3) : 10-12.	程玉莲 李玉兰 钟士鋆 孙玉芳 薛霆. IKAP 模式护理干预对提高社区老年高血压患者自我保健的作用. 齐鲁护理杂志 2008 年 第 03 期
RCT037	Liu ZL, Zhang BL, Wang LX, Xu HP, Wu YS, Mo WH. Observation on the effectiveness Of health promotion and intervention to hypertensive patients in a community. Journal of Clinical Internal Medicine 2008,916(7) : 438-440.	刘梓廉 张冰玲 王丽香 徐汉平 伍运生 莫文华. 社区高血压患者的健康促进与干预效果观察. 临床内科杂志 2008 年 第 09 期
RCT037a	Zhang BL, Liu ZL, Wang LX. Study on the intervention to community primary hypertension and unhealthy living behaviors. Journal of Community Medicine 2008,6(7) : 7-8.	张冰玲 刘梓廉 王丽香. 社区原发性高血压患者与不良生活方式的健康干预. 社区医学杂志 2008 年 第 07 期
RCT038	Li Y, He JF, Jia SF. Influence of Community nursing intervention on hypertensive patients. China Modern Doctor 2008,46(17) : 4-5.	李瑛 何佳峰 贾叙锋. 社区护理干预对高血压患者的影响. 中国现代医生 2008 年 第 17 期
RCT039	Yang X, Liu PW. Observation on the effectiveness of health education in community to result of treatment on hypertensive patients. Shanghai Journal of Preventive Medicine 2008,20(9) : 461-463.	杨欣 刘丕卫. 社区健康教育对高血压患者治疗效果观察. 上海预防医学杂志 2008 年 第 09 期

RCT040	Li PZ, Wu X, Yan L, Li AF. Study on self-management and health education of behavior intervention on hypertensive patients in a Community. Today Nurse (specialized edition) 2008, (5) : 70-77.	李培珍 吴熹 严丽 李岸芳. 社区高血压病自我管理和健康教育行为干预. 当代护士(专科版) 2008 年 第 05 期
RCT041	Zhu YZ. Appraisal to community intervention on hypertension. Journal of Zhejiang College of Traditional Medicine 2008,32(2) : 177-178.	朱益忠. 高血压的社区干预效果评价. 浙江中医药大学学报 2008 年 第 02 期
RCT042	Zeng YM, Deng WB, Xie JJ, Luo K. A study on comprehensive intervention for hypertension in communities. Hainan Medicine 2008,19(8) : 6-7.	曾宇明 邓文彬 谢俊杰 罗科. 高血压病社区综合防治研究. 海南医学 2008 年 第 08 期
RCT043	Wei XR, Fan WH, Yao HQ, Zhang Y. Effectiveness of general practitioner team intervention for uncontrolled hypertension. Shanghai Medicine 2008,31(8) : 579-581.	魏心蓉 范维琥 姚慧青 张颖. 全科团队强化医疗模式干预血压未控制的原发性高血压患者的疗效. 上海医学 2008 年 第 08 期
RCT044	.Xu YL,He PP,Wang J, Shi M. Study on the effectiveness of community nursing intervention to KAP of patients with Hypertension. Shanghai Nursing 2008,8(1) : 36-39.	徐筱莉 贺蓓蓓 王佳 施敏. 高血压患者知信行社区护理干预方法与效果研究. 上海护理 2008 年 第 01 期
RCT045	Yang SC, Zhou F. Approach and appraisal of hypertension-community prevention and control experience of Shuanglong community in Luohe city. China Modern Doctor 2008,46(32) : 131-132, 140.	杨水长 周芳. 漯河市双龙社区高血压综合防治经验探讨及效果评价. 中国现代医生 2008 年 第 32 期
RCT046	Chen WH. Discussion on community nursing comprehensive intervention to the hypertensive patients. Family Nurse 2008, 6(7C) : 1914-1916.	陈伟华. 应用护理程序对社区高血压人群综合干预的探讨. 家庭护士 2008 年 第 21 期
RCT047	Yang LJ, Sui J, Li R, et al. A study on health education to enhance treatment compliance of medicine taking of primary hypertensive patients. Chinese Journal of Health Education, 2008,24(5): 375-376.	杨丽娟,隋捷,李荣,等.健康教育对原发性高血压患者服药依从性的影响[J]. 中国健康教育,2008,24(5):375-376.
RCT048	Su WQ. Discuss on the function of holistic family intervention in community intervention of hypertension. Chinese Journal of Modern Nursing. 2008, 14(14):1541-1543.	苏婉霞. 家庭整体干预在高血压病社区干预中的作用[J] . 中华现代护理杂志, 2008, 14(14) : 1541

RCT049	Wang JJ, Zhu LM, Wang LF, et al. Improving medication-taking compliance and hypertension control rates by health education in hypertension patients. Applied Journal of General Practice. 2008, 6(6):607-609.	王建骏 朱理敏 王丽芬. 健康教育提高社区高血压患者服药依从性和高血压控制率. 实用全科医学 2008 年 第 06 期
RCT050	Xie JQ. Influence of health education on hypertension control rate about young and middle-aged patients. Today Nurse.2009, (11):93-94.	谢俊琴. 健康教育对中青年高血压控制率的影响分析. 当代护士 2009;11(专科版):93-94.
RCT051	Dai XN, Ju RS. Effectiveness of health education on blood pressure control of patients with hypertension. Guide of China Medicine. 2009, 7(23):113-114.	戴贤龙，, 鞠荣生. 社区健康教育对高血压人群血压控制的效果分析. 中国医药指南 2009;7(23):113-114.
RCT052	Gong WL. Observation of effectiveness of health education and intervention in community to primary hypertension. Medical Journal of Chinese People's Health. 2009,21(24):3221,3235..	宫万力. 原发性高血压的社区健康教育和干预效果观察. 中国民康医学 2009 年 第 24 期
RCT053	Hu YC, Huang H, Yang Z, et al. Effects of strengthen intervention of community groups in Nanning city blood pressure with longitudinal follow-up study. Chinese Journal of Geriatric Care. 2009, 7(1):35-39.	胡才友 黄慧 杨泽 吕泽平 李宁 杜治琴. 综合强化干预对血压影响的纵向随访研究 社区人群心脑血管事件前瞻性研究系列之九. 中国老年保健医学 2009 年 第 01 期
RCT053a	Lv ZP, Huang H, Yang Z, et al. Longitudinal follow-up study of changing the risk to cardiovascular events by strengthen intervention of community groups in Nanning city Chinese Journal of Geriatric Care,2009,7(1):18-21.	强化干预代谢综合征改变心脑血管事件发生风险的纵向随访研究 社区人群心脑血管事件前瞻性研究系列之四
RCT053b	Hu CY, Lv ZP, Huang H, et al. Influence study of strengthening intervention hypertension patients with the abnormal glucose metabolism in community population of Nanning City. Journal Of Applied Preventive Medicine. 2009, 15(4):193-197.	胡才友 吕泽平 黄慧 杨泽 李宁 吴军. 强化干预对社区高血压伴糖代谢异常人群心脑血管事件影响研究. 应用预防医学 2009 年 第 04 期
RCT054	Li YL, Liu FL. Influence of intervention in community to hypertension control. Chinese Community Doctor. 2009, 11(24):268.	李颖厉 刘凤玲. 社区干预对高血压控制的效果影响. 中国社区医师(医学专业半月刊) 2009 年 第 24 期
RCT055	Ou YQ. Evaluation on the management of hypertension patients in community nursing interfere Journal of Tongling Vocational & Technical College. 2009, 8(1):40-40, 42.	藕跃勤. 社区护理干预对高血压病人管理效果评价. 铜陵职业技术学院学报 2009 年 第 01 期

RCT056	Wang xl, Wang YQ. Impact of nursing intervention in community to treatment effectiveness of primary hypertension. Journal of Yangtze University (Natural Science Edition).2009, 6(3):193-196.	王小莉 王燕青. 社区护理干预对原发性高血压治疗效果的影响. 长江大学学报(自科版)医学卷 2009 年 第 03 期
RCT057	Shu XH, Lin FY. Influence of nursing intervention in community to treatment effectiveness of patients with hypertension. Modern Clinical Nursing.2009, 8(7):12-14.	舒小芳 林芳宇. 社区护理干预对高血压患者治疗效果的影响. 现代临床护理 2009 年 第 07 期
RCT058	Wei SH, Huang FZ, Ye XM, et al. Influence of nursing intervention in community to older patients with primary hypertension. Family Nurse. 2009,(2):100-101.	韦素惠 黄凤珍 叶小梅. 社区护理干预对老年原发性高血压病病人的影响. 全科护理 2009 年 第 02 期
RCT059	Feng YC, He M, Guo JL, et al. Influence of health education in community on medication adherence of patients with hypertension and treatment evaluation. Hainan Medical Journal. 2009, 20(7):341-342.	冯银彩 何敏 郭劲莲 蔡小玲. 社区健康教育对高血压患者治疗依从性的影响及疗效评价. 海南医学 2009 年 第 S4 期
RCT060	Wang FY. Health education role on treatment efficacy of hypertension in community. Chinese Journal Of Cardiovascular Rehabilitation Medicine. 2009, 18(4):353-354..	王福彦. 社区健康教育对高血压患者血压控制的影响. 心血管康复医学杂志 2009 年 第 04 期
RCT061	Yao LF. Clinical observation of 38 patients with hypertension who received intervention in community and usual medication. Tianjin Pharmacy. 2009, 21(6):36-37.	姚立枫. 社区干预配合常规用药防治高血压 38 例临床观察. 天津药学 2009 年 第 06 期
RCT062	Yang GY. Observation of effectiveness of integrated Prevention and control work for patients with primary hypertension. Guide of China Medicine. 2009, 07(8):49, 7.	杨冠英. 对原发性高血压患者进行社区综合防治的效果观察. 中国医药指南 2009 年 第 08 期
RCT063	Li ZM, Chen JX, Guo S, et al. Community intervention and effect valuation in the young hypertension people. Journal of North China Coal Medical College.2009, 11(6):799-801.	李壮苗 陈锦秀 郭实 刘芳 李霞 张文霞. 高血压低龄群体的社区管理干预与效果评价. 华北煤炭医学院学报 2009 年 第 06 期

RCT064	Lan YJ. Evaluation on effectiveness of intervention in community for patients with hypertension. Guide of China Medicine. 2009. 7(9):247-248. Li XZ. Nursing intervention of effect in patients with hypertension. China Modern Doctor. 2009,47(2):108-109.	蓝玉娟. 高血压患者社区干预的效果评价. 中国医药指南 2009 年 第 09 期
RCT065	Li XZ. Nursing Intervention of Effect in Patients with Hypertension. China Modern Doctor. 2009, 47(2): 108-109.	李小珍. 高血压的社区护理干预效果分析. 中国现代医生 2009 年 第 02 期
RCT066	Zhu L, Wu KM, Ling F, et al. Evaluation of the effectiveness of the group interference-management model for hypertension. Shanghai Journal Of Preventive Medicine. 2009, 21(6):253-256.	朱兰 吴克明 凌枫 高俊岭 刘德安. 上海市某社区高血压群组干预管理效果评价. 上海预防医学杂志 2009 年 第 06 期
RCT067	Wang H, Deng YM. Function of health education for treatment of hypertension. Modern Preventive Medicine. 2009, 36(3):485-488.	王辉 邓咏梅. 健康教育在高血压病治疗中的作用. 现代预防医学 2009 年 第 03 期
RCT068	Yan XQ, Zhang HZ, Deng SL, et al. Impact of community-based health education to family members on the patients with essential hypertension both in the way of life and compliance with prescription. Journal Of Qilu Nursing.2009, 15(7):22-23.	严秀群 张惠珍 邓淑玲 宋小苑. 家属社区家庭健康教育对高血压患者生活方式及服药依从性的影响. 齐鲁护理杂志 2009 年 第 07 期
RCT069	Zeng AR, Wan FY. Impact of nursing intervention to patients with hypertension in community. China Foreign Medical Treatment. 2009, 28(34):131-132.	曾爱蓉 万凤媛. 护理干预对社区高血压患者的影响. 中外医疗 2009 年 第 34 期
RCT070	Xia GH, Lu GJ, Yang ZX. Effectiveness evaluation of health education on aged patients with essential hypertension in one community in Guangzhou. China health education. 2009,25(7):517-518.	夏国华 卢国坚 杨政雄. 广州市某社区老年原发性高血压患者健康教育效果分析. 中国健康教育 2009 年 第 07 期
RCT071	Liu F. Nursing measures on elderly patients with hypertension in communities. Chinese Journal of Ethnomedicine and Ethnopharmacy. 2009;14;147-148.	刘芳. 浅谈社区高血压老年患者护理措施. 中国民族民间医药 2009 年 第 14 期
RCT071a	Liu F. Nursing measures on elderly patients with hypertension in communities. Chinese Journal of Ethnomedicine and Ethnopharmacy. 2009;18(7)：147—148．	刘芳．浅谈社区高血压老年患者护理措施[J]． 中国民族民间医药，2009，18(7)147—148．

RCT072	Lu XE. Effects of nursing intervention in community on HBP patients. Chinese journal of practical medicine. 2009; 22(3):165-166.	卢喜娥．社区护理干预对高血压患者血压的影响[J]． 中华实用中西医杂志，2009，22(3)：165—166．
RCT073	Zhang SY. Nursing intervention on the prognosis of patients with hypertension. China Foreign Medical Treatment. Clinical Medicine. 2009;25:47-48.	张淑英. 护理干预队高血压患者转归的影响. 临床医学, 2009, 25: 47 - 48.
RCT074	Wang JH, Li QH. Influence of two different health education styles on hypertension patients in community. Chinese General Nursing. 2009;7(10):2727-2728.	王家惠, 李清华. 两种健康教育方式对社区高血压病病人血压控制的影响. 全科护理 2009;7(10):2727-2728.
RCT075 (PubMed)	Mendis S, Johnston SC, Fan W, Oladapo O, Cameron A, Faramawi MF. Cardiovascular risk management and its impact on hypertension control in primary care in low-resource settings: a cluster-randomized trial. Bulletin of the World Health Organization 2009;88(6):412-419.	Mendis S, Johnston SC, Fan W, Oladapo O, Cameron A, Faramawi MF. Cardiovascular risk management and its impact on hypertension control in primary care in low-resource settings: a cluster-randomized trial. Bulletin of the World Health Organization 2009;88(6):412-419.
RCT076	Ye Q, Shen XY, Peng LH. Effects of community-based intervention on patients with hypertension. Journal of community medicine. 2010;8(1):13-14.	叶勤 沈小云 彭利花. 高血压病社区干预的效果观察. 社区医学杂志 2010 年 第 01 期
RCT077	Tan XJ. Evaluation on the community management of hypertension. Jilin Medical Journal. 2010;31(21) :3525-3526.	谭新姣. 高血压社区管理疗效评价. 吉林医学 2010 年 第 21 期
RCT078	Zhang D, Li HR, Zhang CX. Effect of comprehensive intervention on Essential hypertension in Changqing Garden Community. 2010; 13(3A):768-769.	张枬 李红荣 张彩霞. 武汉市常青花园原发性高血压病社区综合性干预的效果评价. 中国全科医学 2010 年 第 07 期
RCT078a	Zhang D, Zhou JH. Effect of community-based comprehensive intervention on hypertension. Chinese Community Doctors. 2010;12(19):264-265.	张枬 周建华. 高血压社区综合性干预效果观察. 中国社区医师(医学专业) 2010 年 第 19 期
RCT079	He WJ, Sun XY, Wang Qi. The effect of the community nursing intervention of the treatment compliance on controlling blood pressure in old patients with essential hypertension. Nursing Practice and Research. 2010;7(12):11-13.	何文静 孙晓宇 王琪. 遵医行为的社区护理干预对老年高血压病患者血压控制的影响. 护理实践与研究 2010 年 第 12 期
RCT080	Cai ZY. Effect of community-based nursing intervention on treatment of the patients with hypertension. Chinese Comnmunity Doctors. 2010;12(21):209-210.	蔡志阳. 社区护理干预对高血压患者治疗效果的影响. 中国社区医师(医学专业) 2010 年 第 21 期

RCT081	Guo N, Wu XJ, Wei L, et al. Influence of nursing intervention on essential hypertension among science researchers. Nursing Practice and research. 2010;7(16):18-19.	郭娜 吴欣娟 魏丽 韦春玲 安俊芳. 护理干预对科研人员原发性高血压病患者的影响. 护理实践与研究 2010 年 第 16 期
RCT082	Wang MR, Gui Y, Jiang XJ, et al. The effect of the community nursing intervention of the treatment compliance on controlling blood pressure in patients with essential hypertension. Journal of University of South China(Medical Edition). 2010;38(2):289-290.	王美蓉 桂英 将小剑 奉永利. 遵医行为的护理干预对社区高血压病患者血压控制的影响. 南华大学学报(医学版) 2010 年 第 02 期
RCT083	Huang LX, Wu QX, Zhang QJ, et al. Impact of community nursing intervention on blood pressure of patients with hypertension. Journal of Bengbu Medical College. 2010;(35)3:307-308.	黄丽霞 吴秋霞 张秋娟 张添招. 社区护理干预对高血压患者血压的影响. 蚌埠医学院学报 2010 年 第 03 期
RCT084	Zhang YQ. Influence of community nursing intervention on the compliance of patients with essential hypertension. Chinese General Nursing. 2010;8(4A):932-933.	张艳清. 社区护理干预对原发性高血压病病人遵医依从性的影响. 全科护理 2010 年 第 10 期
RCT085	Zhu HJ. Effect of community-based comprehensive intervention on hypertension control. Prevention and Treatment of Cardio-cerebral-vascular Disease. 2010;10(1):57-58.	朱红君. 社区综合干预控制高血压病效果观察. 心脑血管病防治 2010 年 第 01 期
RCT086	Cai ZH. Effect of community-based compliance intervention on hypertension treatment. Chinese Community Doctors. 2010;12(1):137-138.	蔡正华. 社区高血压病治疗依从性干预效果观察. 中国社区医师(医学专业) 2010 年 第 01 期
RCT087	Li CL, Chen Y, Zhang DX, et al. Evaluation on different hypertension management model. Chinese Journal of Public Health. 2010;26(3):309-310.	李春霖 陈嫣 张东霞 张飞 张萍 郝申强. 不同社区高血压患者管理模式干预效果评价. 中国公共卫生 2010 年 第 03 期
RCT088	Bao XH, Wang CR, Yan Y. Study on the risk factors of hypertension by community-based intervention. Modern Prevention Medicine. 2010;37(4):674-675.	包晓红 王翠蓉 阎妍. 高血压病危险因素的社区干预研究. 现代预防医学 2010 年 第 04 期
RCT089	Peng SQ. Influence of family-centred intervention on compliance of patients with hypertension. Modern Medicine and Health. 2010;26(14):2206-2207.	彭思庆. 以家庭为中心的干预对高血压患者治疗依从性的影响. 现代医药卫生 2010 年 第 14 期

RCT090	Qin XH. A study of the community nursing intervention on improving the compliance behaviour of patients with hypertension. 2010;8(1B):98-99.	覃小慧. 社区护理干预对提高高血压病病人遵医行为依从性的研究. 全科护理 2010 年 第 02 期
RCT091	Xia W, Liu YQ. Evaluation of community-based intervention on hypertension. Journal of Medical Forum. 2010;31(7):51-52.	夏炜 刘玉清. 高血压的社区干预效果评价. 医药论坛杂志 2010 年 第 07 期
RCT092	Yang X, Yang DJ, Yang X. Investigation on status of the fourth medical behaviour and effect of community health service intervention on patients with hypertension. Chinese Journal of hypertension. 2005;13(1):48-50.	阳晓，杨定姣，阳旭．高血压患者第四医学行为状况及社区卫生服务性干预效果的调查[J]．高血压杂志。2005，13(1)：48—50．
RCT093	Wang HJ. Evaluation on health education in primary treatment of essential hypertension. Chinese Community Doctors. 2007;9(4):87.	王浩瑾. 健康教育在基层原发性高血压病治疗中的效果评价[J] . 中国社区医师, 2007, 9 (4) : 87.
RCT094	Sun ZJ, Zhang QB, Ye YL, et al. Effect evaluation of the fourth medical behaviour of patient with hypertension in a community. Modern Preventive Medicine. 2008;35(19):3717-3718.	孙宗建 张青碧 叶运莉 甘仲霖 张元松. 某社区高血压患者第四医学行为社区干预效果评价. 现代预防医学 2008 年 第 19 期
