# Supplementary material for: Effectiveness of interventions for hypertension care in the community – a meta-analysis of controlled studies in China
Source: BMC Health Serv Res. 2012 Jul 24;12:216. doi: 10.1186/1472-6963-12-216 (PMC3416668; doi:10.1186/1472-6963-12-216)
Supplement: Additional file 5 — Main characteristics of included studies –hypertension care in the community in China. [file 1472-6963-12-216-S5.pdf]

Additional File 5: Main characteristics of included studies –hypertension care in the community in China

Study code	Year	Sample size	Follow up (year)	Region	Participants	Age	Male (%)	Intervention	Provider
RCT001	1999	200	0.33	Sichuan	Hypertensive outpatients	40-75	43%	Health education: self-help educational booklets; weekly telephone follow up advice; monthly home visit and group sessions; group discussions	Doctor
RCT002	2000	360	2	Guangdong	Pre-hypertension in old people (60 and above years old)	Mean 66, range 60-79	56.10%	CB interventions: health education (weekly counselling, monthly workshops, self-help materials). Advices on exercise, diet, life style changes, psychological health. Weekly home visit and invovlement of family members.	Nurse
RCT003	2000	334	3	Guangdong	Hypertensive old patients in community	≥60	39.20%	CB comprehensive intervention: patient registry, regular monitoring, services at neighbourhood centres, individual advices, health education (various forms), training community health workers.	Nurse
RCT004	2001	148	0.33	Guangxi	Hypertensive patients in community	35-75	49.30%	CB intensive health education: self-help booklet, monthly workshop and discussion, with biopsy samples, educational videos. BP knowledge competitions every 3 months.	Nurse
RCT005	2002	243	3	Zhejiang	Hypertensive patients in community	Mean 59-63	45.30%	Monthly GP home visits for 3 years and monthly educational workshops: scheduled management of patients with high BP, educational and behavioural interventions	Doctor
RCT006	2002	68	1.5	Zhejiang	Hypertensive patients (not specified)	Mean 52.8 (6.3)	45.70%	CB comprehensive nursing care: educational group or individual sessions, self-help educational materials, relaxation exercise, advice on life-style, exercise, and treatment compliance.	Nurse
RCT007	2003	245	0.33	Hebei	Hypertensive patients from a factory	Mean 63.4 (5.5)	46.10%	Training for self-management: based on self-efficacy theory; group training session led by patient volunteers and supported with professionals; weekly session for 7 weeks.	Unclear
RCT008	2004	180	NR	Guangdong	Hypertensive patients in community	Mean 67	70.60%	Educational interventions: workshops or other educational materials. Advice on life-style and behavioural changes, self-management, and risk and treatment of hypertension.	Unclear

Study code	Year	Sample size	Follow up (year)	Region	Participants	Age	Male (%)	Intervention	Provider
RCT009	2004	104	0.5	Shandong	Hypertensive patients discharged from a hospital	Mean 50 (4), range 47-60	55.80%	Community nursing care: weekly telephone follow up. Advice on family support, self-monitoring, and treatment compliance.	Nurse
RCT010	2004	581	2	Guangdong	Hypertensive patients in community	61.65 (8.27), range 35-81	NR	Community nursing care: monthly home visit for 2 years. Individualised counselling or group sessions; advice on diet, exercise, and psychological health.	Doctor
RCT011	2004	90	2	Beijing	Hypertensive outpatients	66.5 (4.7), range 60-79	100%	Community nursing care: special nursing team, regular daily community visits, educational workshops, healthcare plan. Advice on diet, low salt, life-styles, and treatment compliance.	Nurse
RCT012	2004	219	0.5	Shanghai	Hypertensive patients in community	67.2 (9.5), range 35-80	51.60%	CB self-management: 6 weekly health education sessions for self-management, plus individualised support by telephone couselling (by trained community doctors), and involvement of community.	Doctor
RCT013	2004	400	3	Zhejiang	Hypertensive patients in community	45-82	41.30%	CB management: health education - booklets, group sessions; face-to-face counselling. Advice on diet, medication, life-styles, psychological health, and treatment medications.	Doctor
RCT014	2005	247	4	Shanxi	Hypertensive patients in community	NR	45%	CB comprehensive intervention: monthly home visit for 4 years; advice on behavioural, dietary and psychological health.	Doctor
RCT015	2005	200	2	Zhejiang	Hypertensive patients in community	NR	NR	CB interventions: educational workshops (every three months). Assigned community doctor-patient contacts, monthly BP measurement. Advice on behavioural, psychological health. Individualised pharmacological interventions.	Doctor
RCT016	2005	204	1	Guangdong	Hypertensive outpatients	Mean 52 (range 34 -80)	57.40%	Community nursing care: two home visits during the first 2 months, then at least 2 telephone contacts; advice on self monitoring, family support, life-styles and treatment compliance.	Nurse
RCT017	2005	251	1	Anhui	Hypertensive patients in community	Mean 58.2 (11.5)	57.60%	Family-centred interventions: interview family members to identify and change life styles; neighbourhood intervention to improve family relations. 1-2 home visits per month, and then every 1-3 months. Advice on diet, exercise, psychological health, treatment compliance.	Doctor

Study code	Year	Sample size	Follow up (year)	Region	Participants	Age	Male (%)	Intervention	Provider
RCT018	2005	376	0.5	Guangdong	Old hypertensive patients in community	Old patients (age unclear)	53.20%	Health education: educational workshop every two weeks for 6 months, involving patients and family members. Advice on life style, diet, and knowledge on HBP complications and treatment compliance.	Nurse
RCT019	2006	1000	1	Jiangsu	Hypertensive patients from community	>35	60%	Systematic management of hypertensive patients, according to schedule use of anti-hypertensives (NAH scheme). Monthly monitoring follow-up. Regular health education workshops.	Doctor
RCT020	2006	40	0.5	Shanghai	Hypertensive outpatients in a community hospital	55-65	55%	Regular monitoring follow-up (2-4 outpatient visits per month, plus monthly home visit). Advice on life-style, behavioural and dietary changes, and treatment compliance.	Doctor
RCT021	2006	100	0.083	Anhui	Hypertensive patients in community	48-78	44%	CB health education: self-help booklet, weekly monitoring for 4 weeks, 2 educational workshops. Advice on hypertension risk, and treatment compliance.	Nurse
RCT022	**2006**	108	2	Jiangsu	Hypertensive patients in community	Mean 65.3	55.60%	CB treatment compliance intervention: face-to-face counselling during outpatient visit, intensive and frequent advices on treatment compliance and life-style changes.	Doctor
RCT023	2006	90	3	Guangdong	Hypertensive patients in community	Mean 68 (8.6)	70%	Community nursing care: patient registry, health education -regular community workshops. Advice on diet, exercise, psychological health and relaxation.	Nurse
RCT024	2006	137	1	Beijing	Hypertensive patients in community	NR	48.90%	Health education: 3 formal counselling sessions; individualised health education advice on hypertension knowledge, diet, exercise.	Doctor
RCT025	2007	206	NR	Hebei	Hypertensive patients in community	NR	NR	CB interventions: Regular follow up visits (home or telephone contacts). Individually assigned community doctors to patients. Advice on life-style and behavioural changes, psychological health, and treatment compliance.	Doctor
RCT026	2007	220	1	Jiangsu	Hypertensive patients in community	Mean 49	53.20%	CB multiple modes health education, self-help booklets, face-to-face, family members involved. Advice on risk of hypertension, life-style and behavioural health, diet and treatment compliance.	Doctor
RCT027	2007	87	1	Sichuan	Hypertensive patients - outpatients	Mean 55.2 (15.6)	56.30%	CB - outpatient visits, telephone or home visit follow up, health education workshops, individualized advice on treatment compliance.	Doctor

Study code	Year	Sample size	Follow up (year)	Region	Participants	Age	Male (%)	Intervention	Provider
RCT028	2007	120	0.5	Hunan	Hypertensive patients in community	41-82	65%	Community nursing, weekly telephone follow up, monthly home visit. Advice on life-style, behaviours, diet, psychological health, family support, and treatment compliance.	Nurse
RCT029	2007	56	NR	Zhejiang	Hypertensive patients - outpatients	Mean 66.8 (3.6), 60-78	57.10%	Community nursing, weekly home visit. Advice on family support, self-management, and treatment compliance.	Nurse
RCT030	2007	120	0.5	Hunan	Hypertensive patients discharged from a hospital	Mean 51.8 (5.3); range 45-59	61.70%	Community nursing: advice/material on self-management; frequent telephone/home visit follow up (weekly for one month and then monthly for 5 months). Advice on self-management, life-styles, diet, and treatment compliance.	Nurse
RCT031	2007	120	0.5	Hubei	Hypertensive patients discharged from a hospital	38-73	50.80%	Community health education: 2 group sessions per month, individual counselling of selected patients; plus telephone contacts. Advice on psychological health, life-style, diet, exercise, self BP measurement, and treatment compliance.	Both: Nurse & Doctor
RCT032	2007	200	NR	Hubei	Hypertensive patients in community	Mean 63.8 (2.5)	60%	Health education +family-based interventions: training family members on knowledge of hypertension, life styles and behaviours, and psychological health. Weekly free BP measurement, and appropriate adjustment of medications.	Nurse
RCT033	2007	300	1	Zhejiang (Rural)	Hypertensive patients in community	≥ 60	43%	Health education: every 2 month face-to-face advices; home visit or telephone contact. Advice on hypertension knowledge, life-style, diet, exercise, psychological health, regular BP monitoring, treatment compliance.	Unclear
RCT034	2007	76	0.5	Shandong	Hypertensive patients in community (>60)	60 -74	53.80%	Community nursing care: including community health workers, neighbourhood staff and family members. Health education, advice on diet, exercise, psychological relaxing training, frequent BP monitoring.	Nurse
RCT035	2007	280	1	Zhejiang	Hypertensive outpatients in a community hospital	NR	NR	Community nursing care: various health educational activities (self-help materials, group sessions and patient led discussions). Advice on psychological health, life styles, and treatment compliance.	Nurse

Study code	Year	Sample size	Follow up (year)	Region	Participants	Age	Male (%)	Intervention	Provider
RCT036	2008	272	1	Shandong	Hypertensive patients in community	60-86	61.80%	IKAP nursing model (information, knowledge, attitude, practice): deciding targets according to patient baseline data; providing community nursing care (twice per month for 12 months); advice on life-style changes, self-management, family support, and treatment compliance.	Nurse
RCT037	2008	888	3	Guangdong	Hypertensive patients in community	Mean 62 (6.4); 35 - 75	40.50%	CB health promotion: workshops and other health educational activities; monthly free BP measurement at community centres or at home. Individualised management of hypertensive patients. Advice on life-style changes, and treatment compliance.	Doctor
RCT038	2008	240	0.5	Sichuan	Hypertensive patients discharged from a hospital	Mean 67.8 (4.0), range 58-81	51.70%	Community nursing care: 2 home visit or telephone contacts during the first month, self-help educational information, Individualised advice on risk, prevention and treatment of hypertension, psychological health, life-style, diet, exercise, family support, and treatment compliance.	Nurse
RCT039	2008	354	0.94	Shanghai	Hypertensive patients in community	Mean 68.5 (12.9)	NR	CB health education: Monthly educational workshops, self-help booklets, monthly telephone or outpatient follow up. Advice on psychological health, life-style, and treatment compliance.	Doctor
RCT040	2008	185	0.25	Guangxi	Hypertensive patients in community	NR	NR	Self-management: three monthly training sessions for self-management; self-help booklet; health professionals' support/advice.	Nurse
RCT041	2008	417	NR	Zhejiang (Rural)	Hypertensive patients in community	NR	53.20%	CB intensive health education: monthly group sessions, self-help materials, home visits. Advice on life-styles, psychological health, exercise, low salt, treatment compliance.	Doctor
RCT042	2008	150	3	Guangdong	Hypertensive inpatients discharged from hospital	Mean 66.03 (7.4)	38.70%	CB comprehensive intervention: 1-2 group educational sessions per month, self-help educational materials, regular follow up and monitoring; advices on life style, diet, psychological health, and treatment compliance.	Doctor
RCT043	2008	300	1	Shanghai	Patients with uncontrolled hypertension	31-88	60%	CB primary care intervention: stable relation between doctor and patient; weekly clinic or home visit (or monthly after adequate BP control). Advices on life style, diet, health behaviours, risk of hypertension, and treatment compliance.	Both: Nurse & Doctor

Study code	Year	Sample size	Follow up (year)	Region	Participants	Age	Male (%)	Intervention	Provider
RCT044	2008	130	1	Shanghai	Hypertensive patients in community	50-80	NR	Community nursing: development of a nursing care team; group education sessions (every 3 months); individual face-to-face counselling, regular home or telephone visits; care records, self-help handbooks, family support. Advice on risk factors, medication, diet, physical health.	Nurse
RCT045	2008	386	3	Henan	Hypertensive patients from various sources	Mean 45 (16), range 17-78	49.70%	CB comprehensive intervention: health education (various forms) - workshops every 3 months. Patient and family registry; family support, telephone or home visit.	Doctor
RCT046	2008	120	1	Anhui	Hypertensive patients in community	Mean 62.8	55.80%	Community nursing: risk assessment, action plan agreed between community nurse and patient; implementation of interventions (dietary, physical activities, mental health, compliance to treatment); and evaluations	Nurse
RCT047	2008	200	0.5	Guangdong	Hypertensive outpatients	32-82	62%	Health education: self-help educational materials; workshops and discussion; monthly telephone follow up and advice on treatment compliance.	Nurse
RCT048	2008	106	1	Guangxi	Hypertensive patients in community	Mean 50	52.80%	Family centred interventions: Community nurse home visit intervention for 6 months; family health assessment, health education to family members; behaviour changes plan at home; support from family members.	Nurse
RCT049	2009	402	0.7	Shanghai	Hypertensive patients in community	NR	NR	Health education: 4 workshops in 2 months, advice on BP measurement, risk of hypertension and treatment compliance.	Doctor
RCT050	2009	176	0.5	Hunan	Hypertensive patients	45.5 (30-55)	NR	Health education: individualized, specific and problem orientated. Advice on the risk of hypertension, life-style changes, psychological health, and treatment compliance.	Nurse
RCT051	2009	786	1	Jiangsu	Hypertensive patients in community	≥ 35	NR	Health education: regular advice on the prevention and treatment of high blood pressure to patients in the intervention group (not further details).	Unclear
RCT052	2009	148	1	Jilin	Hypertensive outpatients in a community hospital	62 (5), range 35-88	64.90%	Health education: advice on life-style, behavioural and dietary changes, and treatment compliance	Nurse

Study code	Year	Sample size	Follow up (year)	Region	Participants	Age	Male (%)	Intervention	Provider
RCT053	2009	306	3	Guangxi	Hypertensive patients among community residents (mainly civil servants)	Mean 56.17 (7.93)	52.20%	Intensive health education: Educational workshops, educational booklets. Training of community health workers. Regular follow up monitoring (community, home or telephone). Advice on life-style and behavioural changes.	Doctor
RCT054	2009	106	0.5	Guangxi	Hypertensive patients in community	58.5 (42 -74)	56.60%	CB health education: advice on life-style and behavioural changes, diet, psychological health, and treatment compliance.	Unclear
RCT055	2009	60	1	Anhui	Hypertensive patients in community	60-80	50%	Community nursing acre: regular educational workshops, self-help educational materials, individualised counselling. Advice on life-style, diet, psychological health, exercises, and treatment compliance.	Nurse
RCT056	2009	64	1	Hubei	Hypertensive patients in community	65.3 (35-78)	54.70%	Community nursing care: weekly home visit, regular workshops, self-help educational material. Advice on risk of hypertension, treatment compliance, life-style, diet, self-measurement, and treatment compliance.	Nurse
RCT057	2009	700	2	Guangdong	Hypertensive patients in community	Mean 67.3/66.2 (9.4/8.7); range 50-80	30%	Community nursing car: advice on self-management, diet, exercise, psychological health, and treatment compliance.	Nurse
RCT058	2009	122	1	Guangxi	Hypertensive patients in community	Mean 65.7 (9.9)	51.60%	Community nursing care: home visit twice per month, regular telephone contacts, and outpatient visit arrangement. Advice on risk of hypertension, self-management, life-style, diet, psychological health, and treatment compliance.	Both: Nurse & Doctor
RCT059	2009	640	1.5	Guangdong	Hypertensive patients in community	33-89	44.80%	Community health education: telephone/home visit, workshops, multi-media VCD. Advice on life-style, behaviours, diet, exercise, psychological health, self-BP measurement, and treatment compliance.	Nurse
RCT060	2009	990	1	Zhejiang	Hypertensive patients identified by community screening	NR	NR	CB health education: regular (but no more details) advice on High BP knowledge, life style, diet, and treatment of hypertension.	Nurse
RCT061	2009	76	0.5	Tianjin	Hypertensive patients in community	55.6 (35-78)	55.30%	CB health education (lack of details): advice on diet, low salt, exercise, psychological health, regular self-monitoring, appropriate anti-hypertensive medication.	Unclear

Study code	Year	Sample size	Follow up (year)	Region	Participants	Age	Male (%)	Intervention	Provider
RCT062	2009	200	NR	Guangdong (Rural)	Hypertensive patients in community	66.05 (7.35)	45%	CB Health education: regular group or individual sessions; home visit or telephone follow up, advice on psychological health, diet, exercise, and hypertension treatment.	Unclear
RCT063	2009	110	1	Fujian	Young hypertensive patients in community	Mean 29.4 (3.4), range 23-35	65.50%	CB comprehensive management: patient registry, risk assessment based hypertension management, regular follow up (telephone or home visits -weekly or monthly or every 3 months), educational workshops, weekly free BP measurement. Advice on life style, diet, psychological health, and anti-hypertensive medication.	Unclear
RCT064	2009	100	1	Liaoning	Hypertensive patients in community	NR	NR	CB interventions: patient registry, educational workshops, advice on life style, diet, low salt, psychological health, BP measurement, treatment compliance.	Unclear
RCT065	2009	200	NR	Guangdong	Hypertensive patients (unclear source)	Mean 54.2 (11.3)	60%	Community nursing: BP measurement every two weeks by the assigned nurses.	Nurse
RCT066	2009	369	0.5	Shanghai	Hypertensive patients in community	Mean 70.01 (9.76), range 35-80	36.30%	CB intervention: 7 workshops (every 2 weeks in 3 months; including discussion and individual advices). Advice based on Chinese Guidelines on hypertension management.	Both: Nurse & Doctor
RCT067	2009	366	0.25	Sichuan	Newly diagnosed hypertension outpatients	18-75	52.50%	Health education: individual face-to-face counselling, self-help materials, weekly telephone follow up, outpatient vist every two weeks	Unclear
RCT068	2009	120	0.5	Guangdong	Hypertensive patients in a reseach institute	Mean 54 (11), range 50-81	60.80%	Family-centred health education: Involving both patients and families. Monthly group session, self-help materials; advice on regular BP monitoring, life-styles, hypertension risk and management.	Unclear
RCT069	2009	120	NR	Jiangxi	Hypertensive patients in community	48-82	61.70%	Community nursing care: patient registry, regular telephone contacts, educational workshops, self-help materials. Advices on life styles, regular monitoring, diet, psychological health, and treatment compliance.	Nurse
RCT070	2009	130	1	Guangdong	Old hypertensive patients in community	Mean 63.0 (6.0)	55.40%	Health education: group sessions (every 2 months), self-help booklets, individual advices on medications, diet, physical activities, psychological health, life styles.	Unclear

Study code	Year	Sample size	Follow up (year)	Region	Participants	Age	Male (%)	Intervention	Provider
RCT071	2009	80	0.5	Jiangsu	Old hypertensive patients in community	60-74	54%	Community nursing: health education (group sessions, advices, discussions, self-help booklets). Advice on psychological health, diet, exercise, family support, and treatment compliance.	Nurse
RCT072	2009	220	NR	Hunan	Hypertensive patients in community	61-93	55.50%	Community nursing: health education (group sessions, individual counselling, discussions, self-help booklets). Advice on psychological health, diet, exercise and medications.	Nurse
RCT073	2009	80	0.5	Shangdong	Newly diagnosed hypertension outpatients	30-60	82.50%	Nursing interventions: weekly BP follow up measurement; advice on behaviour changes and treatment compliance.	Nurse
RCT074	2009	120	0.5	Sichuan	Hypertensive patients in community	40-80	55.80%	Health education: community nurses providing individualised health educations, weekly home visits, telephone contacts, and workshops.	Nurse
RCT075	2010	1209	1	Unclear	Newly diagnosed hypertension outpatients	Mean 54 (8.5); 30-70	47%	Training of community doctors to follow WHO protocol for CVD risk management using hypertension as an entry point in primary care. Training workshops, followed by training session 2-4 months later to reinforce the intervention.	Doctor
RCT076	2010	186	2	Jiangxi	Hypertensive patients	Mean 58/60	55.90%	CB managing hypertensive patients: Educational workshops, advice on life-style and behavioural changes, psychological health, and treatment compliance.	Doctor
RCT077	2010	600	1	Guangdong	Hypertensive patients	Mean 56 (34-82)	56%	Educational workshops (once/2 weeks for 14 weeks) for self-management, life-style changes, psychological health, and treatment compliance.	Doctor
RCT078	2010	402	1	Hubei	Hypertensive patients	>35; mean 49 (6.8)	NR	CB management of hypertensive patients: Educational community workshops (2/month), weekly walk-in counselling, home visits or telephone contacts, educational booklets. Advice on risks of hypertension, life-style and behavioural changes, and treatment compliance	Doctor
RCT079	2010	198	0.5	Guangdong	Hypertensive patients discharged from a hospital	60-80	59.60%	Community-nursing care: education, monitoring follow-up monthly for 6 months, facilitate patient self-management	Nurse

Study code	Year	Sample size	Follow up (year)	Region	Participants	Age	Male (%)	Intervention	Provider
RCT080	2010	160	0.83	Hunan	Hypertensive patients discharged from a hospital	45-88	59.30%	Community-nursing: health education, regular monitoring follow-up for 10 months. Support patient self-management, provide individualised advice.	Nurse
RCT081	2010	240	3	Hebei	Hypertensive patients in a research institute	Mean 55.4 (3.6); range 41-70	79.60%	Regular monitoring follow-up, weekly BP measurement, educational workshops (monthly). Advice on psychological health, life-style changes, family support, and treatment compliance.	Doctor
RCT082	2010	78	0.5	Hunan	Hypertensive patients discharged from a hospital	32-65	59.00%	Community nursing care: monthly home visit for 6 months; advice on life style, diet, self-monitoring, family support, and treatment compliance.	Nurse
RCT083	2010	100	0.5	Guangdon	Hypertensive patients in community	38-79	54.00%	Community nursing care: establishing patient record system, frequent telephone/home visit follow up; health education: advice on psychological, behavioural and nutritional health, and treatment compliance.	Nurse
RCT084	2010	100	0.5	Guangxi	Hypertensive outpatients	43-75	62%	Community nursing care: health education, advice on high BP knowledge, life styles, diet, low salt intake, exercise, psychological health, self monitoring, and treatment compliance.	Nurse
RCT085	2010	168	0.5	Zhejiang	Hypertensive outpatients	Mean 65.5 (7.9), range 45-88	54.80%	CB comprehensive intervention: treatment plan agreed by both patient and doctor; fortnight home visit; life style changes, and advice on diet, low salt intake, and psychological health	Doctor
RCT086	2010	152	1	Sichuan	Hypertensive patients in community	63.9 (36 - 82)	58.60%	Community educational & behavioural interventions: before monthly BP measurement, providing face-to-face advice (15 min) on diet, life-style, mental health, exercise, family support, self-monitoring, and treatment compliance.	Doctor
RCT087	2010	413	0.5	Shanghai	Hypertensive patients in community	55.6 (14.7), 35-75	51.10%	CB Self-management: 6 weekly training sessions for self-management; plus self-help booklet, and individualised support/advice.	Doctor
RCT088	2010	214	2	Hubei	Hypertensive patients in community	Mean 62.7	50.90%	CB interventions: education workshops (every 3 months), self-help booklets. Advice on life style, exercise, psychological health, and treatment compliance.	Unclear

Study code	Year	Sample size	Follow up (year)	Region	Participants	Age	Male (%)	Intervention	Provider
RCT089	2010	80	0.33	Hunan	Hypertensive outpatients	NR	NR	Family-centred, professional supported interventions: frequent home and telephone contacts (every 1-3 weeks), involving family members, individualised advice on diet, exercise and life style changes, family support, self-management.	Both: Nurse & Doctor
RCT090	2010	186	0.25	Guangdong	Hypertensive patients discharged from a hospital	45-84	53.80%	Community nursing intervention: patient registry, telephone follow up and advices on health life styles, regular monitoring.	Nurse
RCT091	2010	510	5	Henan	Hypertensive patients in community	NR	52.40%	CB comprehensive management: patient registry. Monthly educational workshops, self-help booklets, home visit. Advice on life style changes, exercise, diet, psychological health, and treatment compliance.	Doctor
RCT092	2005	824	0.5	Hunan	Hypertensive patients in community	58 (35 - 89)	65.80%	CB health education: individual counselling, education posters, self-help materials. Advice on life-style, diet, exercise, psychological health, self-monitoring, family support, and treatment compliance.	Unclear
RCT093	2007	156	0.5	Henan	Hypertensive outpatients	35 -72	55.80%	Health education: individual counselling, advice on life style changes, diet, low salt intake, exercise, psyhcological health, family support, and treatment compliance.	Unclear
RCT094	2008	442	0.5	Sichuan	Hypertensive patients in community	54 (32 - 86)	63.80%	CB interventions: identify patient information need, design educational targets. Home visit, and individual counselling (10-20 minutes) until the target achieved. Advice on life style, diet, exercise, psychological health, and treatment compliance.	Doctor
